# Cervical Cancer Screening in HIV-Positive Farmers in South Africa: Mixed-Method Assessment

**DOI:** 10.5334/aogh.37

**Published:** 2019-04-15

**Authors:** Molly Lieber, Omara Afzal, Kathryn Shaia, Adrienne Mandelberger, Christine Du Preez, Ann Marie Beddoe

**Affiliations:** 1 Icahn School of Medicine at Mount Sinai, US; 2 Hoedspruit Training Trust, ZA

## Abstract

**Background:**

In 2015, a See and Treat cervical cancer screening program was implemented at a local HIV clinic in Limpopo, South Africa, where infrastructure limited adequate Pap smear usability.

**Objectives:**

The purpose of this evaluation was to determine the quality and sustainability of the implemented program.

**Methods:**

A mixed-methods program analysis was conducted at 18-months post implementation. Data collection techniques included in-depth interviews of staff and patients, observation of healthcare workers delivering screening, and review of charts and patient logs.

**Findings:**

Eighteen in-depth interviews revealed improved cervical cancer screening understanding and awareness. Privacy concerns and negative perceptions of medical care were barriers to screening. Informal observations revealed continued clinical competence among healthcare workers who had been previously trained. Review of charts demonstrated positive correlation between VIA and Pap smear results. In evaluating loss to attrition, about half of the first cohort of patients were lost to follow-up. VIAs and Pap smears were offered on an ongoing basis, and month-over-month change for overlapping four months of programming between 2015 and 2016 showed a 4.4% negative change in number of Pap smears and a 57% negative change in VIAs.

**Conclusion:**

Our evaluation reveals successful integration of See and Treat into current clinic services in rural South Africa and increased awareness of cervical cancer among health workers and participants. Program sustainability was challenging to assess as many patients were lost to follow-up, given the migrant and transient population attending this clinic. Acceptance by health workers and patients alike is vital for the long-term impact on cervical cancer incidence in this region.

## Introduction

Each year, 528,000 cases of cervical cancer are diagnosed worldwide, and cervical cancer accounts for 266,000 deaths [1]. While the incidence and mortality have decreased in high-income countries, they remain high in less-developed regions, including Southern Africa. In 2012, South Africa had a cervical cancer incidence of 7,735 per 100,000, and mortality was 4,248 per 100,000 [1]. Although human papilloma virus (HPV) vaccines are available to prevent cervical cancer, financial and political barriers contribute to their underutilization and inaccessibility. These barriers contribute to women in lower-resource populations seeking care for this cancer late in the course of their disease when symptoms occur. The disease burden is high, and outcomes are poor [2,3]. Consequently, a large emphasis is put on cervical cancer screening. Traditional cytology-based screening, such as Pap smears, has been successful in reducing cervical cancer rates in high-income countries, but it is less accessible and efficacious in low-income countries due to the requirement for robust infrastructure [4]. Specifically, challenges in rolling out cytology-based screening include lack of trained health workers, availability of appropriate equipment, finances, and logistics for follow-up care [2,4,5].

As a result, many low-income settings have adopted a See and Treat method for cervical cancer screening [4,6,7]. The See and Treat method, which includes visual inspection with acetic acid (VIA) for diagnosis and cryotherapy for treatment, has been recommended by the World Health Organization (WHO) for low-resource settings and for populations where women may be lost to follow-up [8]. Serving as a low-tech screening method, VIA involves application of acetic acid to the cervix and subsequent visual inspection [4]. Results are available immediately, and treatment can be offered at the time of screening to avoid multiple patient visits and possible loss to follow-up [4,7,9]. Because shortages of physicians are historic barriers to providing care in low-resource and rural areas, the See and Treat method has been proposed for these settings as trained healthcare workers can be readily trained to perform both VIA and cryotherapy procedures [10,11]. Results from previous studies show that nurses are able to master the numerous steps involved in a See and Treat program, including recruitment, education, and performance of clinical exams and treatment [12]. While the See and Treat method is the best option for many settings, there are limitations, including procuring and transporting cryotherapy machines and gas tanks, refilling tanks, and repairing damaged cryotherapy machines [13].

Although there is no consensus about the length of training and education needed for VIA training, most programs agree that both didactic and clinical training components are essential. Previous studies show varied lengths of training and education, ranging from two days to two weeks [4,5], and programs developed from varying organizations often include didactic lectures, simulation, and clinical practice [2]. One review showed that after 10 days of training, nurses agreed with experts’ VIA assessments more than 60% of the time [16].

The major problem with VIA is that quality assurance and quality improvement have not been standardized, and various methods have been used to assess consistent health worker performance and overall success of See and Treat programs. Confirmation of VIA results through review of VIA photographs by gynecologists and medical officers, confirmation of diagnosis through web-based instruments, and follow-up visits by initial program implementers have all been utilized to assist with program monitoring and evaluation [5,17]. Repeat trainings and periodic refreshers have been shown to increase average test scores and confirm or improve clinical practices [18].

In developing countries where HIV rates are high, the double burden of HIV and HPV infections makes integration of VIA screening services ideal as access to target populations is secured and sustainability of programming is increased [10]. For HIV positive populations, cervical cancer screening is of utmost importance, as there is an increased risk of invasive cervical cancer among HIV positive women [15]. This known association occurs for many reasons, including similar risk factors for HPV infection and HIV positive populations and higher susceptibility to HPV infection among HIV positive populations [14]. More rapid progression and higher rates of cervical intraepithelial neoplasia are seen among HPV and HIV co-infected individuals, and disease progression is not affected by use of anti-retroviral therapy (ART). HIV positive individuals accessing ART have higher life expectancy and thus higher cumulative risk for HPV infection [14].

In 2015, in Limpopo, South Africa, we introduced a cervical cancer screening program in an existing HIV screening and treatment clinic servicing HIV positive migrant farm workers and sex workers [19]. The South Africa National Policy for cervical cancer screening recommends three Pap smears in a lifetime. As discussed separately, chart reviews revealed poor Pap smear quality, long delays in addressing abnormal results, and many lost results [20], initiating our introduction of See and Treat cervical cancer screening. In order to determine the quality and sustainability of the program, we conducted an evaluation utilizing a mixed-method approach at 18 months post-implementation. Using a mixed-method approach, an outcome-based evaluation was designed with the primary objective of assessing whether the program was sustained and functional. The secondary objective was to determine next steps to improve quality of the program in the future.

## Methods

### The Program

**Figure 1:**
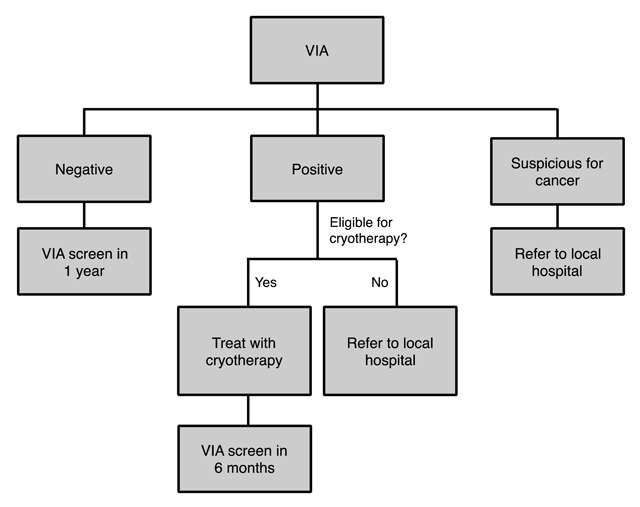
Algorithm used in current program.

The initial program design followed the World Health Organization VIA guidelines and was adjusted for this population; it was implemented in 2015 as previously reported [19]. Researchers conducted a one-week training and spent three months overseeing implementation of the program. At the end of three months, trained health workers continued to run the program and educate additional nursing staff using the “train the trainer” model. Program participants were female patients who attended the HIV clinics; they were offered screening for cervical cancer using VIA and cryotherapy if screening results were positive. The implemented algorithm included six-month follow-up post VIA to troubleshoot and reinforce procedures and guidelines (Figure 1).

Our aims were to (i) assess advancements in knowledge and skills of providers and improvements in knowledge, attitudes, and behaviors of participants; (ii) assess perspectives and experiences of participants and providers; and (iii) determine gaps in the program that would inform overall necessary improvements moving forward. Institutional review board approval was obtained from both the United States and South Africa.

### Site

The evaluation was conducted at a farm-based HIV clinic in Limpopo Province, South Africa, where cervical cancer screening was introduced 18 months earlier. Participants were recruited from the clinic and included health providers, ancillary health workers, and patients. Our measurement tools included interviews, focus groups, clinical observation, review of charts, and review of clinic logs.

Qualitative interviews were completed using a field guide of 12 questions. Questions were developed to illicit responses regarding acceptability of cervical cancer screening, particularly VIA, among health workers and patients. Additionally, questions focused on understanding of screening, knowledge of cervical cancer and HPV, and barriers to receiving reproductive healthcare. Interviewees were recruited at the clinic using convenience sampling over the course of five days. All nurses and community health workers spoke English and acted as interpreters for patients whose first language was not English.

One focus group was conducted with nurses to assess their perspectives and experiences with the addition of cervical cancer screening into their routine HIV counseling and treatment program.

Health workers who underwent initial training in cervical cancer screening were observed by the ObGyn team from the United States during the course of their activities. Providers were observed during patient interviews, speculum examinations, and VIA performance and graded as “poor,” “fair,” or “good.”

Quantitative data, Pap smear and VIA results, and cryotherapy was obtained from chart reviews and patient logs. Charts of the first patient cohort (N = 403), those screened at program rollout, were reviewed and logs were evaluated 18 months post initial program integration.

Qualitative data was recorded and transcribed by research team members and was coded to elicit themes. SPSS and Microsoft Excel were used for analysis of data.

## Results

### Interviews

Eighteen in-depth interviews were conducted with patients (12) and healthcare providers, including counselors (3), nurses (1), and peer educators known as nompilos (2). One focus group of nurses was conducted. The focus group revealed high levels of cervical cancer screening understanding and awareness, as well as privacy concerns and negative perceptions of medical care as barriers to screening.

### Cervical Cancer Screening and Treatment: Comfort and Challenges

Acceptability by Health Workers. Nurses expressed a sense of empowerment for their acquired See and Treat skills. One nurse conveyed pride when discussing her ability to heal patients at the clinic.

“If had not been seen, sent to hospital. Now, can help them right away, straight away.” (Community Health Worker)

Comfort. In discussing comfort level within the concept of screening and the actual screening process, patients discussed their experiences and nurses considered the perceived comfort of patients. Most referenced a discomfort with the position required for undergoing screening, and that comfort increased with each visit. Additionally, patients mentioned feeling more at ease with explanations from the nurses at the clinic.

“First time, tool they’re using is uncomfortable. Nervous about getting results. Cryo, afraid at first. Can’t see what’s going on her own body. New and unseen.” (Patient)

“At first she was afraid she thought maybe she would have pains, and she asked them and they said no it’s not painful and now she’s comfortable.” (Patient)

Anatomy and Purpose: Patients expressed understanding of Pap smears and VIA screening in terms of both anatomy and purpose. Most were able to identify cervical cancer as the disease being screened for and understood the importance of screening.

“The most important thing is that if you go for cervical cancer and then you’re still on the first stage, early stage, you will get help. Yea, it’s very important to get it on early stage before it spread.” (Patient)

“If you got infection, they will get it earlier than when it’s too late and you will get help here if it’s not too much, you will get it in the early stage.” (Patient)

“Because cancer is a dangerous disease. If you don’t go for a check then you go later then you can die.” (Patient)

Privacy As a Challenge. When asked about challenges to screening, nurses referenced patients’ concerns about privacy, specifically referencing a feeling of older patients not wanting to be seen by younger nurses.

“About 10–20% refuse Pap smear because they are too nervous or concerned with privacy. Some have fear and don’t want to ask questions. They don’t want to be taught, even at meetings or churches.” (Community Health Worker)

“Largest challenge, patients don’t want be seen. Older women didn’t want young nurses to see her. Not comfortable with the position and being seen.” (Community Health Worker)

Negative Perceptions of Medical Care As a Challenge. Medical services are lacking in this area of South Africa as the hospital is understaffed and has a high rate of turnover among top administrators and lower level workers. Consequently, when patients seek care at the hospital, they wait for many hours, possibly days, they are uninformed about practices and procedures, and they are often left behind in follow-up care. Nurses and patients alike cited many of these issues as reasons for having negative perceptions of medical care.

“Some of the people they say I’m going home I don’t want to go there because maybe they will cut me something, so they go home.” (Patient)

“Largest challenge for patients to receive treatment at the hospital—they have to wait many hours just to book an appointment. Sometimes the hospital doesn’t book them. Most of the patients don’t wind up going.” (Community Health Worker)

### Training and Education Approach

Train the Trainer: Patients and nurses expressed satisfaction with the train the trainer method of education. With their current network of clinics, they are able to provide additional nurses with hands-on training after the original trainers left.

“They sometimes teach at the clinic they used to teach everyone. Yes, because if the other people they don’t know maybe they would be happy to know now.” (Patient)

Challenges. Nurses expressed a desire for continuous education as they were unclear how to move forward when facing challenges they were unfamiliar with.

“[We] need more education. Challenges, unable to help with certain things; couldn’t do VIA. Finally find cervix and os and there is oozing at the top. [I think] who to ask, how do they refer to the hospital. Pap is negative, but VIA looks strange. (Community Health Worker)

Nompilos. Community educators are called nompilos and work to spread HIV prevention and treatment information to the community. Patients expressed gratitude for the current HIV work of nompilos. Additionally, they felt the best way to disseminate information was to go to the people and tell them about it.

“I think it’s helpful like here where I’m working, I’m also spreading the information and people are coming to get help here at [clinic] so it’s helping because we share the information.” (Nompilo)

“Like those care groups walk door to door, they can increase the awareness when they going into communities, spreading that information that contraception is good and yes, you can use it.” (Patient)

“The best is to go to people. Yes, go to people. Try to orient them by maybe doing workshops … telling them the advantages & disadvantages, show them close example.” (Patient)

### Unmet Needs

Patients and the community health workers were asked what gaps they felt existed in the current screening process and what they would like to see in the future. Both groups discussed expansion to other clinics and more education.

More Locations. The nurses expressed a desire to expand the See and Treat approach to other clinics, as the main clinic is the only one currently offering cryotherapy.

“Other clinics in the area do not have cryo.” (Nurse)

“More patients are being referred now, they are telling their families and friends but if they are not HIV+ they can not receive treatment at [clinic]. What will they do? They need cryotherapy at other clinics.” (Nurse)

More education. Health workers and nurses both cited a need and want for more education, specifically about cancer. Patients wanted additional information about cancer, specifically the causes, symptoms, stages, and available treatment options for each scenario.

“If she gets cervical cancer, what happens if she gets pregnant? Can she become pregnant if she has cervical cancer? What would happen?” (Patient)

“What are the symptoms of cancer? What is the cause of the growth? How does it come?” (Patient)

### Observations

Informal observations revealed continued clinical competence among healthcare workers; nurses were able to correctly perform the procedure and triage patients appropriately for treatment without assistance. After training, nurses had improved in speculum insertion and ability to examine the cervix. Assessment of quality and performance showed those who were taught to perform VIA and cryotherapy maintained and continued these skills.

### Chart Review

Validity. As patients routinely undergo both Pap smear and VIA screening, we analyzed the correlation between VIA and Pap smear results. Review of charts demonstrated a positive correlation between VIA and Pap smear results [r = 0.321, n = 82, p = 0.003; r = 0.463, n = 183, p = 0.000] in 2016 and 2015, respectively.

Loss to Attrition. In evaluating loss to attrition (Table 1), about half of the first cohort of patients were lost to follow-up (54.8% of VIA+ patients and 61% of VIA– patients). Of those patients who received treatment, necessitating additional screening, 60% were lost to follow-up. VIAs and Pap smears were offered on an ongoing basis, and month-over-month change for overlapping four months of programming between 2015 and 2016 showed a 4.4% negative change in number of Pap smears and a 57% negative change in VIAs. Clinic records were often incomplete or missing, and thus assessment for percentage seen was not calculable.

**Table 1:** Loss To Attrition.

	T1	T2	T3

	N = 403	N = 114	N = 403

Visual Inspection with Acetic Acid (VIA)			

Yes	403 (100%)	13 (11%)	87 (21.6%)
VIA Positive	124 (30.8%)	4 (3.5%)	7 (8.0%)
Cryotherapy	114 (92%)	–	–
VIA Negative	279 (69.2%)	9 (7.9%)	80 (92%)
Ineligible	0 (0%)	1 (0.9%)	
No	0 (0%)	33 (29%)	79 (19.6%)
Loss to Follow-Up	–	68 (60%)	237 (58.8%)
Pap Smear			

Yes	183 (45.4%)	30 (26.3%)	96 (23.8%)
Pap Positive	49 (12.2%)	11 (36.7%)	14 (14.6%)
Pap Negative	134 (33.3%)	19 (63.3%)	77 (80.0%)
No	199 (49.4%)	21 (18.4%)	57 (14.1%)
Loss to Follow-Up	21 (5.2%)	63 (55.3%)	248 (61.5%)

* VIA is the abbreviation for Visual Inspection with Acetic Acid. ^†^ Time 1 (T1) is program implementation, Time 2 (T2) is 6 month follow-up for those who received cryotherapy at T1, Time 3 (T3) is 1-year follow-up for all those who were screened at T1.

## Conclusion

Our evaluation reveals increased awareness of cervical cancer among health workers and participants and successful integration of See and Treat into a clinic in rural South Africa. Using the See and Treat approach and following guidelines for triaging of positive screens, abnormal results can be readily addressed and treated promptly. The program was maintained and patients were treated on site without additional referrals for treatment. Although patients were lost to follow-up, given the migrant nature of the participants, program sustainability was achieved in that clinic healthcare workers are continuing to perform See and Treat exams and awareness of cervical cancer is increasing. Other factors that affect the implementation of the See and Treat program include HIV treatment policy changes made by the South African government. Specifically, HIV treatment is now offered to all HIV-positive individuals, regardless of immune system status (CD4 cell count), which previously determined eligibility for treatment. Consequently, other programs that do not fit in line specifically with HIV treatment may take a back seat while this new policy is enacted. HIV-affected individuals are a recognized unique population, often with needs and challenges that affect multiple aspects of their social and health status, which may play into the feasibility of implementing a widespread cervical cancer screening program.

Limitations include loss to follow-up based on participant status as migrant farm workers. As a result, 237 of the original 403 participants who were screened using VIA (59%) were lost to follow-up. Moreover, as is common in many under-resourced settings, accuracy and consistency in record keeping was lacking, and as is common with document review, content was unreliable and possibly biased. Additionally, turnover among staff at the local clinic was a factor. The main nurse administrator who took on leading the implementation of the See and Treat method left shortly after program implementation. As a result, there is less organization of women undergoing cervical cancer screening. Additionally, the clinic is now short-staffed because filling a nurse’s position is difficult. Even with these challenges, successful See and Treat exams are ongoing.

Interviews with patients and health care providers showed interest and growing understanding of cervical cancer screening and implications on health care. Interviews conducted with the use of a translator may possibly have been subject to bias or limited patient response given the provider-patient relationship. However, the community health workers have been trained in objective and ethical research practices prior to interviewing, and patients appeared open and frank with their answers, which was encouraged. Acceptance by health workers and patients alike is vital for the long-term impact on cervical cancer incidence in this region. Although loss to follow-up occurred, the success of this program can be seen with its continuation and increased public awareness of cervical cancer by patients and health care workers alike.

The results of this assessment will inform ongoing cervical cancer screening programs in low-resource settings and with similar patient populations by highlighting the successes and barriers that must be addressed for successful and widespread program implementation. Given the new guidelines for HPV screening and the awareness that has been created regarding the role of HPV in cervical cancer in this region, transitioning to Screen and Treat is now being considered. Using HPV screening with triage to VIA in the same setting will allow us not only to widen our screening coverage, but also to readily treat patients where the migrant nature of the population accounts for high rates of loss to follow-up.

## Data Accessibility Statement

All authors had access to data and a role in writing this manuscript.
